# Performance of Large Language Models in Recognizing Brain MRI Sequences: A Comparative Analysis of ChatGPT-4o, Claude 4 Opus, and Gemini 2.5 Pro

**DOI:** 10.3390/diagnostics15151919

**Published:** 2025-07-30

**Authors:** Ali Salbas, Rasit Eren Buyuktoka

**Affiliations:** 1 Department of Radiology, Izmir Katip Celebi University, Ataturk Training and Research Hospital, Izmir 35150, Turkey; 2 Department of Radiology, Foca State Hospital, Izmir 35680, Turkey; rasiterenbuyuktoka@hotmail.com

**Keywords:** large language models, magnetic resonance imaging, MRI sequences, hallucination, artificial intelligence, susceptibility-weighted imaging, brain MRI

## Abstract

**Background/Objectives**: Multimodal large language models (LLMs) are increasingly used in radiology. However, their ability to recognize fundamental imaging features, including modality, anatomical region, imaging plane, contrast-enhancement status, and particularly specific magnetic resonance imaging (MRI) sequences, remains underexplored. This study aims to evaluate and compare the performance of three advanced multimodal LLMs (ChatGPT-4o, Claude 4 Opus, and Gemini 2.5 Pro) in classifying brain MRI sequences. **Methods**: A total of 130 brain MRI images from adult patients without pathological findings were used, representing 13 standard MRI series. Models were tested using zero-shot prompts for identifying modality, anatomical region, imaging plane, contrast-enhancement status, and MRI sequence. Accuracy was calculated, and differences among models were analyzed using Cochran’s Q test and McNemar test with Bonferroni correction. **Results**: ChatGPT-4o and Gemini 2.5 Pro achieved 100% accuracy in identifying the imaging plane and 98.46% in identifying contrast-enhancement status. MRI sequence classification accuracy was 97.7% for ChatGPT-4o, 93.1% for Gemini 2.5 Pro, and 73.1% for Claude 4 Opus (*p* < 0.001). The most frequent misclassifications involved fluid-attenuated inversion recovery (FLAIR) sequences, often misclassified as T1-weighted or diffusion-weighted sequences. Claude 4 Opus showed lower accuracy in susceptibility-weighted imaging (SWI) and apparent diffusion coefficient (ADC) sequences. Gemini 2.5 Pro exhibited occasional hallucinations, including irrelevant clinical details such as “hypoglycemia” and “Susac syndrome.” **Conclusions**: Multimodal LLMs demonstrate high accuracy in basic MRI recognition tasks but vary significantly in specific sequence classification tasks. Hallucinations emphasize caution in clinical use, underlining the need for validation, transparency, and expert oversight.

## 1. Introduction

Large language models (LLMs), following their remarkable success in the field of natural language processing in recent years, have increasingly begun to be used in health informatics and clinical decision support systems [1,2]. Initially limited to text-based information, these models can now process not only textual data but also various types of input, such as images, audio, and even video, thanks to the advent of multimodal versions [3]. In visually intensive disciplines such as radiology, this advancement has the potential to enhance diagnostic accuracy and clinical decision-making, optimize radiologists’ workflows, and enable new approaches in medical image analysis [4,5].

LLMs have been evaluated in various ways within radiology and other medical specialties for both text-based and image-based tasks. These models have demonstrated varying levels of success in tasks such as answering specialty-related questions, summarizing reports, identifying anatomical structures, and detecting a wide range of pathologies [6,7,8,9]. Moreover, multimodal LLMs have recently been tested in clinically critical scenarios such as stroke, brain tumors, and intracranial hemorrhage [9,10,11]. However, it remains unclear whether these models can reliably recognize fundamental image characteristics, such as modality or sequence. In other words, a model that cannot recognize the basic structure of an image cannot be expected to reliably analyze clinically complex scenarios. In this context, the present study aims to comparatively evaluate the performance of three multimodal large language models, namely ChatGPT-4o (OpenAI), Claude 4 Opus (Anthropic), and Gemini 2.5 Pro (Google), in tasks involving modality recognition, plane identification, assessment of contrast-enhancement status, and particularly sequence classification in magnetic resonance imaging (MRI). Existing studies on this topic are limited, typically focusing on a few MRI sequences and evaluating earlier LLM versions [9,12]. In this respect, our study represents the first comprehensive comparative analysis in the English literature to evaluate the discriminative performance of current multimodal LLMs using six different brain MRI sequences.

## 2. Materials and Methods

In this study, the performance of the most up-to-date large language models capable of analyzing visual content, namely ChatGPT-4o (OpenAI, https://chat.openai.com, accessed on 23 June 2025), Gemini 2.5 Pro (Google, https://gemini.google.com, accessed on 23 June 2025), and Claude 4 Opus (Anthropic, https://claude.ai, accessed on 23 June 2025), was compared in regard to their ability to distinguish modality type, anatomical region, imaging plane, contrast-enhancement status, and sequence information from brain MRI images. The images were selected from the hospital’s PACS (Picture Archiving and Communication System) and consisted of scans from adult patients without pathological findings, chosen at random. All images were anonymized and contained no personal data. Institutional ethics committee approval was obtained for the study (date: 19 June 2025; Decision No. 0393). ChatGPT-4o (OpenAI) was used for English language editing, translation, and the generation of certain figures and illustrations. In accordance with current transparency guidelines for the use of generative AI tools in medical research, all AI-assisted content was carefully reviewed and revised by the authors to ensure accuracy and integrity [13].

The MRI images were obtained using two different 1.5 Tesla scanners with similar sequence protocols and acquisition parameters (MAGNETOM, Siemens Healthcare, Erlangen, Germany, and Optima 360, GE, Fairfield, CT, USA). A total of 130 brain MRI images were included in the evaluation, consisting of 10 single-slice images for each of 13 representative MRI series. The MRI series used in the study were as follows: axial T1-weighted (T1w), axial T2-weighted (T2w), axial fluid-attenuated inversion recovery (FLAIR), coronal FLAIR, sagittal FLAIR, coronal T2w, Sagittal T1w, axial susceptibility-weighted imaging (SWI), axial diffusion-weighted imaging (DWI), axial apparent diffusion coefficient (ADC), contrast-enhanced axial T1w, contrast-enhanced coronal T1w, and contrast-enhanced sagittal T1w.

For each MRI series, a single representative slice was selected at an anatomical level, where the lateral ventricles were clearly visible, ensuring that each image reflected the typical visual characteristics of its respective sequence. All images were exported in high-quality JPEG format (minimum resolution: 994 × 1382 pixels), without any compression, cropping, or visual post-processing. No annotations, arrows, or textual markings were present on the images. Original resolution and anatomical proportions were preserved.

Each image was individually uploaded using the official web interfaces of the respective language models, and a standardized English prompt was provided in a zero-shot setting:

“This is a medical research question for evaluation purposes only. Your response will not be used for clinical decision-making. No medical responsibility is implied.

Please examine this medical image and answer the following questions:

1. What type of radiological modality is this examination?
2. Which anatomical region does this examination cover?
3. What is the imaging plane (axial, sagittal, or coronal)?
4. Is this a contrast-enhanced image or not?
5. If this image is an MRI, what is the specific MRI sequence? If it is not an MRI, write ‘Not applicable.’

Please number your answers clearly.”

In order to prevent the models from altering their response strategies based on previous answers within the same session, a phenomenon known as in-context adaptation, a new session was initiated for each prompt by clearing the chat history [14,15]. The reason for presenting the questions in English is that all three models have been primarily trained on English-language content and therefore demonstrate a high level of comprehension and response generation in this language [15,16]. All LLM evaluations were conducted between 23 June 2025 and 29 June 2025, using the most up-to-date versions of the models available at that time. The responses generated by the LLMs were independently reviewed and jointly classified as “correct” or “incorrect” by two radiologists in consensus. Hallucinations were defined as statements unrelated to the input image or prompt context.

### Statistical Analysis

The performance of each LLM was evaluated across five classification tasks based on accuracy, calculated from the number of correct and incorrect responses. Among these, MRI sequence classification was designated as the primary outcome, and formal statistical comparisons were performed only for this task. Differences in model performance for MRI sequence classification were assessed using Cochran’s Q test for overall comparison, followed by pairwise McNemar tests with Bonferroni correction where appropriate. In addition to accuracy, macro-averaged F1 scores and Cohen’s kappa coefficients were calculated to evaluate inter-class performance consistency and agreement with ground truth. To provide stability estimates for sequence-specific accuracy, bootstrap resampling (1000 iterations) was applied, and 95% confidence intervals were reported for each MRI sequence and model.

For the contrast-enhancement classification task, standard binary classification metrics were computed, including sensitivity, specificity, positive predictive value (PPV), negative predictive value (NPV), accuracy, and F1 score, each with corresponding 95% confidence intervals. For the remaining tasks (modality identification, anatomical region recognition, and imaging plane classification), only descriptive statistics (accuracy rates) were reported without formal hypothesis testing, as these were not defined as primary outcomes. The number and distribution of misclassifications across MRI sequences and models were also analyzed. Confusion matrices and error heat maps were generated for each model to display class-specific misclassification patterns. All statistical analyses were performed using SPSS version 28.0 (IBM Corp., Armonk, NY, USA). A *p*-value of <0.05 was considered statistically significant.

## 3. Results

The recognition performance of the ChatGPT-4o, Claude 4 Opus, and Gemini 2.5 Pro models on brain MRI images was evaluated across five distinct tasks, using a total of 130 brain MRI images. All models achieved 100% accuracy in identifying the imaging modality and determining the general anatomical region (brain) (Table 1).

In distinguishing the imaging plane (axial/coronal/sagittal), ChatGPT-4o and Gemini 2.5 Pro achieved 100% accuracy, while Claude 4 Opus demonstrated an accuracy of 99.23%. The only misclassification by the Claude model in this task was a coronal FLAIR image labeled as axial. In the detection of contrast-enhancement status, ChatGPT-4o and Gemini 2.5 Pro achieved an accuracy of 98.46%, while Claude 4 Opus recorded 95.38% (Table 1). In this task, the ChatGPT-4o model correctly identified all 100 non-contrast images as non-contrast, and accurately classified 28 out of 30 contrast-enhanced images, misclassifying the remaining 2. The Claude 4 Opus model also correctly identified all non-contrast images; however, it correctly classified 24 of the contrast-enhanced images and misclassified 6. The Gemini 2.5 Pro model correctly classified 99 out of 100 non-contrast images and misclassified 1, while correctly identifying 29 of the contrast-enhanced images and misclassifying 1. Based on these results, ChatGPT-4o and Gemini 2.5 Pro demonstrated higher sensitivity and comparable specificity in distinguishing between contrast-enhanced and non-contrast images compared to Claude 4 Opus (Table 2).

In the MRI sequence classification task, ChatGPT-4o, Gemini 2.5 Pro, and Claude 4 Opus achieved accuracy rates of 97.69%, 93.08%, and 73.08%, respectively. There was a statistically significant difference in accuracy rates among the models in this task (*p* < 0.001). ChatGPT-4o and Gemini 2.5 Pro demonstrated significantly higher accuracy rates compared to Claude 4 Opus (*p* < 0.001 and *p* < 0.001, respectively). On the other hand, the difference in accuracy between ChatGPT-4o and Gemini 2.5 Pro was not statistically significant (*p* = 0.077).

Statistically significant differences were observed among the accuracy rates of the three models for FLAIR, DWI, ADC, and SWI sequences (Table 3). For FLAIR images, ChatGPT-4o demonstrated significantly higher accuracy compared to Claude 4 Opus (*p* = 0.016); however, no statistically significant differences were found between Claude 4 Opus and Gemini 2.5 Pro (*p* = 1.00), or between Gemini 2.5 Pro and ChatGPT-4o (*p* = 0.070). In the DWI sequence, ChatGPT-4o and Gemini 2.5 Pro had equal accuracy rates, both demonstrating significantly higher accuracy compared to Claude 4 Opus (*p* = 0.031 for both comparisons). Similarly, in the ADC sequence, ChatGPT-4o and Gemini 2.5 Pro showed statistically significantly higher accuracy rates than Claude 4 Opus (*p* = 0.003 for both comparisons). In the SWI sequence, both ChatGPT-4o and Gemini 2.5 Pro achieved 100% accuracy, while Claude 4 Opus exhibited statistically significantly lower performance with 0% accuracy (*p* = 0.002 for both comparisons). Since ChatGPT-4o and Gemini 2.5 Pro demonstrated equal accuracy rates (100%) across all three sequences (DWI, ADC, and SWI), no comparative tests were performed between these two models. For T1- and T2-weighted sequences, all three models achieved 100% accuracy, and thus, statistical comparisons were not required.

In addition to classification accuracy, overall performance metrics for MRI sequence recognition were calculated for each model. These included the macro-averaged F1 score, Cohen’s kappa coefficient, and 95% confidence intervals for accuracy. The results are presented in Table 4. Per-sequence accuracy rates with 95% confidence intervals based on bootstrap resampling are summarized in Table 5.

A total of 47 misclassifications were identified in the sequence recognition task. Of these, 35 were made by Claude 4 Opus, 9 by Gemini 2.5 Pro, and 3 by ChatGPT-4o. Claude 4 Opus classified 10 out of 30 FLAIR images incorrectly as T1-weighted; 9 of these misclassified images were sagittal, and 1 was coronal. Additionally, it misclassified all 10 SWI images and 6 out of 10 DWI images as FLAIR. Out of 10 ADC images, Claude 4 Opus classified 8 as “T2 or FLAIR” and 1 as “T2/FLAIR”. The Gemini 2.5 Pro model misclassified 9 out of 30 FLAIR images; these incorrect responses included 5 classified as DWI, 3 as T1, and 1 as T2. The ChatGPT-4o model misclassified 3 FLAIR images as T1-weighted.

**Figure 1:**
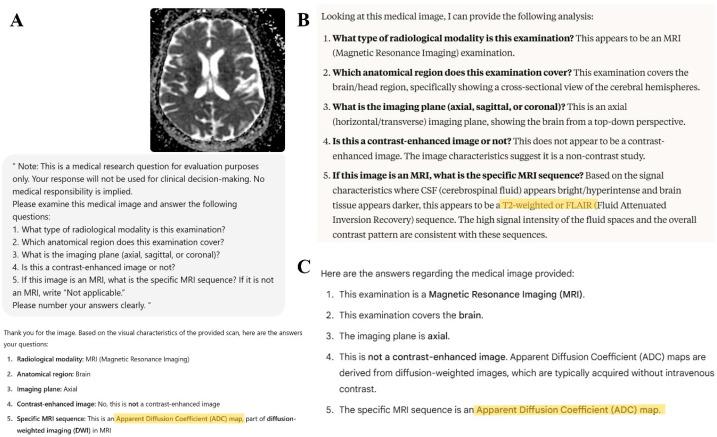
Responses of large language models to an axial brain MRI image corresponding to an ADC map. (**A**) displays the prompt and the correct classification by ChatGPT-4o, which accurately identifies the sequence as an ADC map. In (**B**), Claude 4 Opus misclassifies the same image as either T2-weighted or FLAIR, citing signal characteristics of cerebrospinal fluid and brain tissue. (**C**) The response of Gemini 2.5 Pro, which correctly identifies the sequence as an ADC map. Highlighted text indicates the MRI sequence names as identified by the LLM responses.

**Figure 2:**
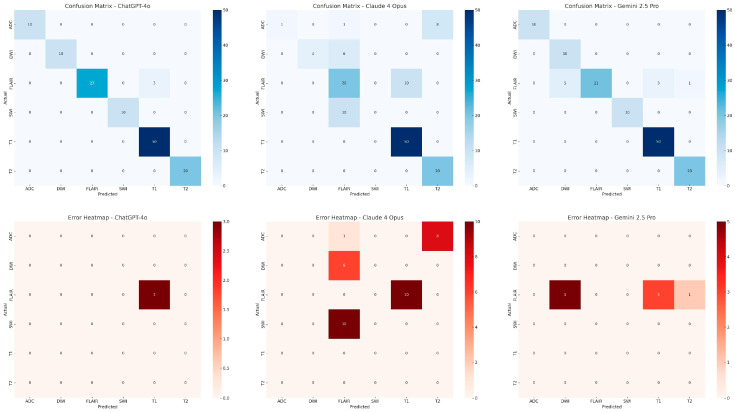
Confusion matrices (top row) and error heatmaps (bottom row) for MRI sequence classification performance of three multimodal large language models: ChatGPT-4o (**left**), Claude 4 Opus (**center**), and Gemini 2.5 Pro (**right**). True labels are on the *y*-axis, and predicted labels on the *x*-axis.

The most frequently misclassified sequence type was FLAIR, which was confused with T1, DWI, and T2 sequences. Misclassifications involving SWI, DWI, and ADC sequences were observed exclusively in the Claude 4 Opus model (Figure 1). Confusion matrices and error heatmaps for all three models are presented in Figure 2.

**Figure 3:**
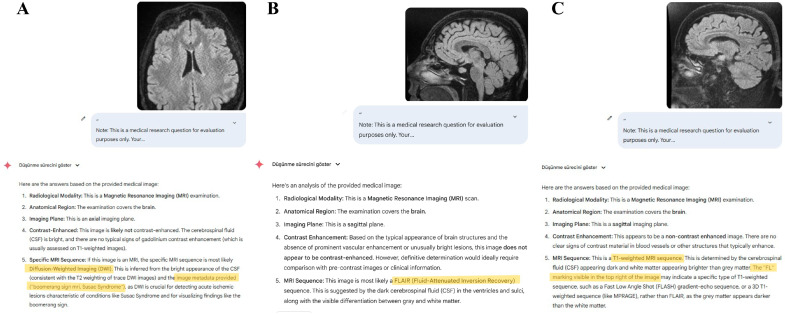
Responses from Gemini 2.5 Pro to three different FLAIR brain MRI images illustrating variability in sequence classification accuracy and response content. In (**A**), an axial FLAIR image is misclassified as a DWI sequence. The response includes additional contextual statements referencing “Susac Syndrome” and the “boomerang sign,” which were not present in the original prompt. In (**B**), a sagittal FLAIR image is correctly identified as FLAIR. In (**C**), a sagittal FLAIR image is incorrectly identified as a T1-weighted sequence. The justification includes mention of an “FL” label that does not exist in the actual image. For display purposes, the prompt is shown in its collapsed form in the figure panels; the full text is identical to the prompt described in the Methods section. Highlighted text indicates the MRI sequence names and/or additional contextual information generated by the LLM responses.

**Figure 4:**
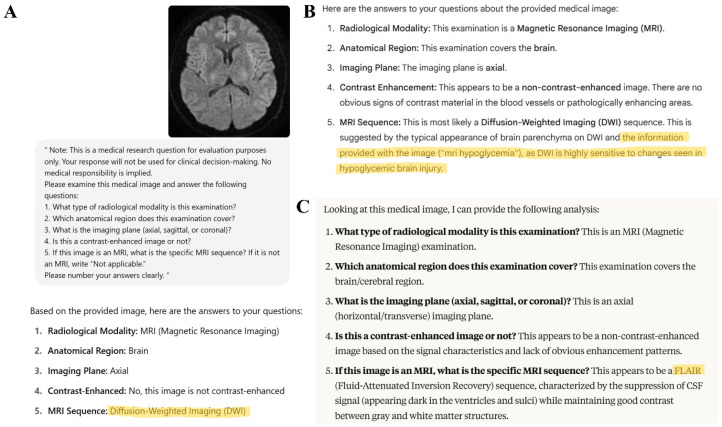
Representative responses from large language models to a brain MRI image corresponding to a DWI sequence. In (**A**), ChatGPT-4o correctly classifies the sequence as DWI. In (**B**), Gemini 2.5 Pro correctly classifies the image as DWI; however, the response includes fabricated contextual information such as “mri hypoglycemia,” which was not present in the original input. (**C**) The response of Claude 4 Opus, which incorrectly identifies the same image as a FLAIR sequence. Highlighted text indicates the MRI sequence names and/or additional contextual information generated by the LLM responses.

In a total of five responses from the Gemini 2.5 Pro model, despite providing only the image and the standardized input, the model was observed to justify its answers using expressions not included in the input. Three of these responses involved incorrect sequence classifications: An axial FLAIR image was misclassified as DWI, with the terms “Susac syndrome” and “boomerang sign” appearing in the model’s response (Figure 3). A sagittal FLAIR image was classified as a T1-weighted sequence based on an “FL” marker that was not present in the image (Figure 3). A coronal FLAIR image was classified as DWI, and the response included unsupported statements such as “normal dwi mri,” which were not present in the original input. In the remaining two examples, although the model correctly identified the DWI images, it included clinical context not provided in the original prompt, such as “hypoglycemia” (Figure 4). Gemini 2.5 Pro exhibited hallucinations in 5 out of 130 responses, corresponding to a rate of 3.8% (95% CI: 1.3–8.7%), while no hallucinations were observed for ChatGPT-4o and Claude 4 Opus.

## 4. Discussion

This study demonstrated that multimodal large language models (LLMs) can achieve high accuracy in basic recognition tasks such as identifying the imaging modality, anatomical region, and imaging plane, with most models reaching or approaching 100% accuracy. However, performance varied more noticeably in complex tasks like MRI sequence classification, where accuracy ranged from 73.1% to 97.7% depending on the model. Claude 4 Opus showed more frequent misclassifications, particularly in FLAIR and SWI sequences, whereas ChatGPT-4o and Gemini 2.5 Pro achieved relatively higher accuracy levels. Additionally, some outputs contained hallucinated statements, raising concerns about model reliability in medical applications.

In our study, all LLMs achieved 100% accuracy in identifying both the imaging modality and the anatomical region in brain MRI images. Similarly, two studies conducted using ChatGPT-4 demonstrated that the model was able to distinguish imaging modalities with 100% accuracy [8,17]. In another study comparing different versions of language models, three versions of ChatGPT and the Claude 3.5 Sonnet model classified CT, MRI, and radiography images with 100% accuracy. On the other hand, the Claude 3 Haiku (87%) and Claude 3 Opus (99%) models misclassified some CT images as MRI, demonstrating relatively lower performance [18]. In the same study, accuracy rates among models for anatomical region identification ranged from 61% to 85%, with the highest accuracy observed for CT images, followed by radiography and MRI images. Similarly, Brin et al. reported that anatomical region recognition accuracy may vary depending on the imaging modality used, achieving up to 100% accuracy for radiography images but dropping to as low as 60.9% for ultrasound (US) images [17]. In the study by Elek et al., Microsoft Bing correctly identified CT modality in abdominal images with 95.4% accuracy and MRI modality with 86.1% accuracy. The accuracy rates for correctly determining anatomical localization were reported as 90% for CT and 77.7% for MRI [19]. Such performance variations may be related to differences both in the LLMs evaluated and the anatomical regions assessed in these studies. The brain region, being well-defined in imaging and relatively difficult to confuse with other anatomical structures, may have contributed to the high accuracy rates observed in modality and anatomical region identification tasks by the models in our study.

Studies evaluating the success of identifying imaging planes (axial, coronal, and sagittal) in radiological images are quite limited in the literature. In abdominal CT and MRI images, Microsoft Bing’s plane recognition accuracy was reported as 95.4% and 83.3%, respectively, with most misclassifications occurring between axial and coronal planes [19]. In contrast, in our study, the ChatGPT-4o and Gemini 2.5 Pro models correctly identified the imaging plane in all brain MRI images, whereas the Claude 4 Opus model demonstrated an accuracy of 99.23%, misclassifying only a single coronal FLAIR image as axial. This discrepancy is likely attributable to the use of brain MRI images without pathological findings in our study and the application of more recent multimodal LLM versions. Images with more standardized visual characteristics may have facilitated the models’ discrimination of imaging planes.

Accurate detection of contrast-enhancement status based on medical images remains a task that has been insufficiently explored in current large language models. In a study conducted on abdominal CT images, Microsoft Bing was reported to identify contrast-enhancement status with an accuracy rate of 64.2% [19]. On the other hand, an open-source, radiomics-based artificial intelligence algorithm developed in 2024, although not LLM-based, achieved considerably high accuracy (90.1%) in classifying contrast phases in abdominal CT images using segmentation and radiomic features [20]. In our study, ChatGPT-4o and Gemini 2.5 Pro achieved an accuracy of 98.46% in detecting contrast-enhancement status, while Claude 4 Opus achieved an accuracy of 95.38%. All three models correctly classified most of the non-contrast images; the majority of misclassifications occurred in classifying contrast-enhanced images as “non-contrast.” These findings indicate that multimodal LLMs can achieve high accuracy in contrast evaluation but still carry a limited margin of misclassification. The high accuracy rates obtained may be associated with the specific anatomical region (brain) and imaging modality (MRI) used in this study. Additionally, the use of the most recent LLM versions may have contributed to this success due to their more advanced visual analysis capabilities. However, it has been reported in the literature that, even when models correctly classify contrast status, they sometimes provide technically incorrect justifications (e.g., “the image is black and white, indicating that no contrast was administered”) [19]. Therefore, LLM responses should be carefully evaluated not only in terms of their outcomes but also with regard to the justifications they provide.

In the current literature, studies evaluating the ability of LLMs to recognize MRI sequences have typically focused on a limited number of sequences and have utilized different model versions (Table 6). A previous study reported accuracy rates of 88.3–90.1% for ChatGPT-4V in a task involving only DWI and ADC sequences [21]. Similarly, Ozenbas et al. reported that ChatGPT-4o achieved an accuracy of 88.3% in brain MRI images involving T1, T2, and FLAIR sequences [9]. In another study involving T1, T2, and FLAIR sequences, the reported accuracy rates were 61.6% for GPT-4o, 46.3% for Gemini 1.5 Pro, and 66.2% for the Grok model. The authors particularly emphasized that FLAIR sequences were identified with lower accuracy and were frequently confused with T2 sequences [12]. In contrast, in our study, ChatGPT-4o, Gemini 2.5 Pro, and Claude 4 Opus classified six different brain MRI sequences with accuracy rates of 97.7%, 93.1%, and 73.1%, respectively. In addition to accuracy, macro-averaged F1 scores and Cohen’s kappa were calculated to provide a more comprehensive assessment of model performance in sequence classification. These measures confirmed that ChatGPT-4o and Gemini 2.5 Pro not only achieved higher accuracy but also demonstrated better inter-class consistency and agreement with ground truth, whereas Claude 4 Opus showed limited reliability across sequence types. It is notable that Claude 4 Opus exhibited a marked performance decline, particularly in FLAIR, DWI, ADC, and SWI sequences. Notably, the Claude 4 Opus model labeled sequences such as ADC and DWI with ambiguous terms like ‘T2 or FLAIR. Among all LLMs, the highest number of misclassifications was observed in FLAIR images, which were frequently misclassified as T1 and DWI sequences.

The FLAIR sequence is a T2-weighted magnetic resonance imaging technique that utilizes long inversion times to suppress cerebrospinal fluid (CSF) signals. Since only normal brain MRI images were used in our study, the partial similarity of the FLAIR sequence with both T1 and DWI sequences in terms of CSF signal characteristics may have complicated the differentiation among these three sequences by LLMs. Additionally, insufficient training data containing adequate variability of FLAIR sequences for some models—particularly Claude 4 Opus—might also be a contributing factor to these misclassifications. This tendency may have been further accentuated by the presentation of only a single representative image slice per case in our study. It is known that Claude’s training is largely based on publicly available internet data, suggesting that the model may lack sufficient exposure to medical imaging datasets [22].

In contrast, ChatGPT-4o and Gemini 2.5 Pro models demonstrated strong performance in sequence differentiation, achieving high accuracy rates. Specifically, sequences such as SWI, previously reported to be identified with lower accuracy, were completely misclassified by Claude, whereas these two models achieved 100% accuracy [23]. In our study, all three models were tested using a standardized zero-shot prompt in separate sessions to ensure consistency and minimize prompt-related bias. Thus, the observed performance differences likely reflect variations in training data exposure, model architecture, and visual reasoning capabilities.

Although Claude 4 Opus is a next-generation LLM equipped with visual analysis capabilities, no published studies directly evaluating its MRI sequence recognition performance currently exist. In a study conducted with text-based questions similar to radiology board examinations, GPT-4 achieved an accuracy of 83.3%, whereas Claude reached only 62% [24]. On the other hand, there are instances where Claude models outperform other LLM models in certain tasks. For example, it has been reported that Claude 3.5 Sonnet exhibited higher accuracy than GPT-4o in evaluating acute stroke using DWI images or provided more consistent responses in certain tasks [10,18]. These differences may arise from task-specific training and architectural characteristics of the models.

**Table 6:** Accuracy of large language models in MRI sequence classification tasks across published studies.

Study	Sequence Types	Image Region	Model	Accuracy (%)
Kuzan et al. [21]	DWI, ADC	Brain MRI (normal and pathologic)	ChatGPT-4V	88.3 (pathologic), 90.1 (normal)
Ozenbaş et al. [9]	T1, T2, FLAIR	Brain MRI (pathologic)	ChatGPT-4o	88.3
Sozer et al. [12]	T1, T2, FLAIR	Brain MRI (normal and pathologic)	ChatGPT-4o	61.67
			Gemini 1.5 Pro	46.38
			Grok Vision Beta (xAI, https://x.ai)	66.23
Elek et al. [19]	T1, T2	Abdominal MRI (pathologic)	Microsoft Bing	68.8
Current Study	T1, T2, FLAIR, DWI, ADC, SWI	Brain MRI (normal)	ChatGPT-4o	97.7
			Gemini 2.5 Pro	93.1
			Claude 4 Opus	73.1

LLMs can occasionally produce unverifiable or input-independent information. This phenomenon is referred to as “hallucination” in the literature [25]. It particularly poses challenges for the applicability and reliability of LLMs in critical fields such as radiology [26]. It has been reported in the literature that LLM-based chatbots can introduce fictitious findings into radiology reports, potentially posing a threat to patient safety [25]. Examples reflecting this tendency were also observed in our study: Although the Gemini 2.5 Pro model correctly classified certain images, it included irrelevant and fabricated clinical information such as “hypoglycemia” or “Susac syndrome,” which were not present in the provided prompt. Such responses should be considered examples of hallucination, which may pose reliability issues from a medical perspective. The “hallucinations” and unnecessary clinical justifications observed in Gemini 2.5 Pro, despite its high accuracy rates, represent a critical concern regarding the clinical reliability of the models. This situation underscores the indispensability of continuous validation, transparency, and human oversight in the integration of LLMs into medical practice. In addition to hallucinations, broader ethical considerations must be addressed when evaluating LLMs for clinical use. These include potential algorithmic bias, lack of transparency about training data origins, and the critical importance of expert oversight. Uncritical reliance on generative AI tools in neuroscience may entail significant risks and ethical concerns, underscoring the importance of human oversight and validation [27]. Therefore, while LLMs hold promise for supporting radiological workflows, their implementation must be guided by rigorous safeguards and human validation.

This study has several limitations. Firstly, only ten single-slice images per sequence were used, all from normal brain MRIs without pathological findings. This limited and homogenous sample may restrict representativeness and lead to an overestimation of model performance. Secondly, analyses were performed using only a single representative image slice, without utilizing three-dimensional or serial images, thus limiting the assessment of model performance based on volumetric information. Furthermore, obtaining images from only two different 1.5 Tesla scanners restricted the evaluation of the potential impact of technical variables such as device manufacturer, magnetic field strength, or imaging protocol. Additionally, the use of a limited number of images restricts the generalizability of our findings. Finally, the LLMs were evaluated using a zero-shot approach, and analyses were based solely on accuracy, thereby limiting a more in-depth investigation into the underlying rationale of the models’ decisions. Despite these limitations, our study is among the first to systematically evaluate the ability of multimodal large language models to distinguish brain MRI sequences, providing a meaningful contribution to the literature. In this context, our study has compared the performance levels of current models in fundamental recognition and discrimination tasks using brain MRI images. Additionally, it sheds light on potential limitations that may be encountered when integrating LLMs into clinical practice. Moreover, future studies may benefit from benchmarking LLMs against dedicated medical imaging models such as convolutional neural networks (CNNs) or vision transformers (ViTs), using standardized public brain MRI datasets. In the future, using larger datasets that include various modalities, sequences, and pathological findings; testing models with multi-slice images; and qualitatively examining the rationale behind model responses will better elucidate the role of LLMs in medical image analysis. Additionally, the development of next-generation LLMs trained on radiological imaging data and optimized for clinical contexts could further accelerate progress in this field.

## 5. Conclusions

This study demonstrated that multimodal large language models generally exhibit high accuracy in basic recognition tasks, such as identifying the imaging modality and anatomical plane in brain MRI images. However, significant performance differences among models were observed in more discriminative tasks, such as sequence classification. This situation should be carefully considered regarding the clinical application of LLMs in critical scenarios. This is particularly important because models may not only inaccurately assess fundamental visual characteristics but also occasionally generate fabricated statements not included in the input, a circumstance requiring caution in terms of safety during complex diagnostic tasks. Therefore, reliable utilization of LLMs in medical image analysis requires a comprehensive approach based not only on accuracy rates but also on the transparency of decision rationales and expert supervision.

## Figures and Tables

**Table 1:** Performance of three large language models on classification tasks, using brain MRI images.

	ChatGPT-4o	Claude 4 Opus	Gemini 2.5 Pro
Modality identification	130/130 (100.00%)	130/130 (100.00%)	130/130 (100.00%)
Anatomical region recognition	130/130 (100.00%)	130/130 (100.00%)	130/130 (100.00%)
Imaging plane classification	130/130 (100.00%)	129/130 (99.23%)	130/130 (100.00%)
Contrast-enhancement status	128/130 (98.46%)	124/130 (95.38%)	128/130 (98.46%)
MRI sequence classification	127/130 (97.69%)	95/130 (73.08%)	121/130 (93.08%)

Note: Each model was evaluated on 130 brain MRI images per task. Values represent the number and percentage of correct responses.

**Table 2:** Performance metrics of large language models in distinguishing contrast-enhanced from non-contrast brain MRI images. Sensitivity, specificity, positive predictive value (PPV), negative predictive value (NPV), and F1 score are reported for each model, along with their corresponding 95% confidence intervals (CIs).

	Sensitivity (95% CI)	Specificity (95% CI)	PPV (95% CI)	NPV (95% CI)	F1 Score
ChatGPT-4o	93.3% (78.7–98.2)	100.0% (96.3–100.0)	100.0% (87.9–100.0)	98.0% (93.1–99.5)	96.6%
Claude 4 Opus	80.0% (62.7–90.5)	100.0% (96.3–100.0)	100.0% (86.2–100.0)	94.3% (88.2–97.4)	88.9%
Gemini 2.5 Pro	96.7% (83.3–99.4)	99.0% (94.6–99.8)	96.7% (83.3–99.4)	99.0% (94.6–99.8)	96.7%

**Table 3:** Classification accuracy of large language models across selected MRI sequence types. Accuracy values are reported as percentages and correct/total counts. *p*-values are reported for rows in which statistical comparison was performed.

Sequence	ChatGPT-4o	Claude 4 Opus	Gemini 2.5 Pro	*p*
FLAIR	90% (27/30)	67% (20/30)	70% (21/30)	0.037
DWI	100% (10/10)	40% (4/10)	100% (10/10)	0.002
ADC	100% (10/10)	10% (1/10)	100% (10/10)	<0.001
SWI	100% (10/10)	0% (0/10)	100% (10/10)	<0.001
T1w and T2w	100% (70/70)	100% (70/70)	100% (70/70)	__

**Table 4:** Overall performance metrics of large language models in brain MRI sequence classification. Accuracy values are reported with 95% confidence intervals. Macro-averaged F1 score and Cohen’s kappa are included to reflect inter-class performance balance and agreement with ground truth.

Metric	ChatGPT-4o	Claude 4 Opus	Gemini 2.5 Pro
Accuracy (%)	97.69%	73.08%	93.08%
95% CI (accuracy)	(93.43–99.21%)	(64.87–79.96%)	(87.37–96.32%)
Macro F1 score	0.9864	0.4085	0.9283
Cohen’s kappa	0.9694	0.6322	0.9089

**Table 5:** Per-sequence accuracy rates and 95% confidence intervals for each multimodal large language model in the MRI sequence classification task. Confidence intervals were estimated using bootstrap resampling (1000 iterations).

Sequence	ChatGPT-4o	Claude 4 Opus	Gemini 2.5 Pro
	Accuracy (95% CI)	Accuracy (95% CI)	Accuracy (95% CI)
T1	100.0% (100.0–100.0)	100.0% (100.0–100.0)	100.0% (100.0–100.0)
T2	100.0% (100.0–100.0)	100.0% (100.0–100.0)	100.0% (100.0–100.0)
FLAIR	90.1% (80.0–100.0)	66.8% (50.0–83.3)	69.9% (53.3–86.7)
DWI	100.0% (100.0–100.0)	39.6% (10.0–70.0)	100.0% (100.0–100.0)
ADC	100.0% (100.0–100.0)	10.1% (0.0–30.0)	100.0% (100.0–100.0)
SWI	100.0% (100.0–100.0)	0.0% (0.0–0.0)	100.0% (100.0–100.0)

Values indicate accuracy (%), followed by 95% confidence intervals calculated using bootstrap resampling (*n* = 1000).

## Data Availability

The dataset supporting the findings of this study has been made publicly available via Figshare and can be accessed at the following link: https://figshare.com/s/6d288a330f6a951ada0a (https://doi.org/10.6084/m9.figshare.29489858, accessed on 7 July 2025).

## Abbreviations

The following abbreviations are used in this manuscript:

LLM	Large language model
CT	Computed Tomography
MRI	Magnetic resonance imaging
T1w	T1-Weighted
T2w	T2-Weighted
FLAIR	Fluid-attenuated inversion recovery
SWI	Susceptibility-weighted imaging
DWI	Diffusion-weighted imaging
ADC	Apparent diffusion coefficient
PACS	Picture Archiving and Communication System
SPSS	Statistical Package for the Social Sciences
PPV	Positive predictive value
NPV	Negative predictive value
CSF	Cerebrospinal fluid
ChatGPT	Chat Generative Pretrained Transformer
CI	Confidence interval
